# Role of Claudins in Renal Branching Morphogenesis

**DOI:** 10.14814/phy2.14492

**Published:** 2020-09-25

**Authors:** Jasmine El Andalousi, Halim Khairallah, Yuan Zhuang, Aimee K. Ryan, Indra R. Gupta

**Affiliations:** ^1^ Research Institute of McGill University Health Centre Montreal Children's Hospital Montréal Québec Canada; ^2^ Department of Human Genetics McGill University Montréal Québec Canada; ^3^ Department of Pediatrics Montreal Children's Hospital McGill University Montréal Québec Canada

**Keywords:** kidney explant, mouse, tight junction

## Abstract

Claudins are a family of tight junction proteins that are expressed during mouse kidney development. They regulate paracellular transport of solutes along the nephron and contribute to the final composition of the urinary filtrate. To understand their roles during development, we used a protein reagent, a truncated version of the *Clostridium perfringens* enterotoxin (C‐CPE), to specifically remove a subset of claudin family members from mouse embryonic kidney explants at embryonic day 12. We observed that treatment with C‐CPE decreased the number and the complexity of ureteric bud tips that formed: there were more single and less bifid ureteric bud tips when compared to control‐treated explants. In addition, C‐CPE‐treated explants exhibited ureteric bud tips with larger lumens when compared to control explants (*p* < .05). Immunofluorescent analysis revealed decreased expression and localization of Claudin‐3, −4, −6, and −8 to tight junctions of ureteric bud tips following treatment with C‐CPE. Interestingly, Claudin‐7 showed higher expression in the basolateral membrane of the ureteric bud lineage and poor localization to the tight junctions of the ureteric bud lineage both in controls and in C‐CPE‐treated explants. Taken together, it appears that claudin proteins may play a role in ureteric bud branching morphogenesis through changes in lumen formation that may affect the efficiency by which ureteric buds emerge and branch.

## Graphical abstract

We demonstrate that removal of multiple claudins from tight junctions within the ureteric bud lineage impairs branching morphogenesis in a mouse embryonic kidney explant model. Fewer ureteric bud tips arise and those that do form are more simple in morphology and they have larger lumens. In summary, claudins regulate renal branching morphogenesis.

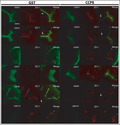

## 1 INTRODUCTION

The process of ureteric bud branching morphogenesis is critical for establishing the location and final number of nephrons that form during kidney development. While much is known about the process of branching morphogenesis including the fact that it is guided by a Turing‐type ligand‐receptor based model in which the ligand, glial‐derived neurotrophic factor, (GDNF) in the mesenchyme binds to and signals to the Ret tyrosine kinase receptor on the ureteric bud (Menshykau et al., 2019) we know little about the cellular events that mediate the process (Costantini, 2012). Ureteric bud cell proliferation is highest in the ureteric bud tips and lowest in the trunks and contributes to the overall growth of the ureteric bud lineage, but does not appear to directly drive the formation of ureteric bud tips (Michael & Davies, 2004). Studies done by Packard et al. demonstrated that ureteric bud tip cells delaminate from the basement membrane and undergo mitosis in the ureteric bud lumen such that one daughter cell reinserts in the original position, while the other reinserts randomly in a position 1–3 cells away (Packard et al., 2013). This process of mitosis‐associated cell dispersal results in extensive cell rearrangements within the ureteric bud tips, but it is not clear if it results in the formation of new ureteric bud tips. Another cellular event that might be important for ureteric bud tip formation and bifurcation is cell shape change: ureteric bud tip cells tend to be more wedge‐shaped with a reduced apical membrane domain when compared to ureteric bud cells within the trunk. Recently, members of the claudin family of tight junction proteins were shown to be important for cell shape changes at the neural plate midline during neural tube closure (Baumholtz et al., 2017).

Claudin mRNA transcripts are detected in a number of expression microarrays and SAGE analyses of the developing rodent kidney (Schmidt‐Ott et al., 2005; Siddiqui et al., 2005; Stuart, Bush, & Nigam, 2003) even before the presence of a urinary filtrate. This intriguing observation suggests that claudins may have additional roles in the developing kidney beyond paracellular transport. In previous work, we explored the roles of claudins during kidney development and determined that a number of claudins are expressed in the developing mouse kidney using RT‐PCR (Haddad et al., 2011; Khairallah et al., 2014). Here, we took advantage of a protein reagent that selectively removes a subset of claudins, Claudin‐3, −4, −6, −7, −8 and −14, from tight junctions without causing any toxic effects. A truncated form of the *Clostridium perfringens* enterotoxin, containing only the C‐terminal domain (C‐CPE), binds to the second extracellular loop of a subset of claudin family members and removes them from tight junctions via internalization of a claudin‐C‐CPE complex (Fujita et al., 2000; Gao & McClane, 2012; Katahira, Inoue, Horiguchi, Matsuda, & Sugimoto, 1997a; Katahira, Sugiyama, et al., 1997; Kimura et al., 2010; Lohrberg et al., 2009; Sonoda et al., 1999; Veshnyakova et al., 2010; Winkler et al., 2009). We treated mouse embryonic explants at embryonic day 12 with C‐CPE and noted that there was a decrease in ureteric bud tip formation compared to control explants. Treatment with C‐CPE also decreased the complexity of branching morphogenesis with more single and fewer bifid ureteric bud tips observed. By immunofluorescence, treatment with C‐CPE led to a decrease in expression of Claudin‐3, −4, −6, and −8 in the apical domain of the ureteric bud tip cells and this correlated with larger ureteric bud tip lumens when compared to control explants. Taken together, it appears that C‐CPE‐sensitive claudins may play a role in ureteric bud branching morphogenesis through changes in lumen formation and in the efficiency of ureteric bud tip bifurcation.

## 2 METHODS

### 2.1 Mouse explant culture

The *Hoxb7/GFP* transgene was backcrossed onto an outbred CD1 background (Srinivas, Goldberg, Watanabe, D'Agati, & al‐ Awqati Costantini, 1999) and *Hoxb7/GFP* ^+/−^ embryos were generated. Kidneys were dissected from *Hoxb7GFP* ^+/−^ embryos at embryonic day 12 and grown in culture as previously reported (Gupta, Lapointe, & Yu, 2003). All mouse husbandry and breeding was performed in accordance with the regulations of the Canadian Council on Animal Care and approved by the McGill University Animal Care Committee (AUP #4120). Embryonic day 12 mouse kidneys were grown in the presence of GST (200 μg/mL) or GST‐C‐CPE (200 μg/mL) for 72 hr in culture.

### 2.2 Production of C‐CPE protein

GST alone and GST fused N‐terminal to the C‐terminal amino acids 185–319 of *Clostridium perfringens* enterotoxin (Moriwaki, Tsukita, & Furuse, 2007) was cloned into pGEX6P1 and then induced by Isopropyl‐1‐thio‐β‐d‐galactopyranoside (IPTG). GST fusion proteins were purified from *E. coli* BL21 strain as described previously (Veshnyakova et al., 2010) and dialyzed against PBS. Protein concentration was determined using the Bio‐Rad Protein Assay (BioRad, Mississauga, Canada).

### 2.3 TUNEL and proliferation assays

The TUNEL assay was performed on explants cultured in the presence of C‐CPE or GST using the In Situ Cell Death Detection Kit, TMR red (cat No. 12 156 792 910; Roche) using Terminal deoxynucleotidyl transferase (TdT) and by adding tetramethylrhodamine (TMR)‐labeled red nucleotides. E12 explants were cultured in the presence of 200 μg/mL of C‐CPE or GST for 72 hr and then fixed in formalin for 1 hr at room temperature and processed for cryosectioning. Slides were washed for 30 min in PBS and then permeabilized with 0.1% triton and 0.1% sodium citrate for 2 min on ice. Slides incubated with DNAse‐1 at a concentration of 3 U/mL for 20 min at room temperature were used as a positive control for the TUNEL assay. Slides were washed two times for 5 min each in PBS and incubated with the labeling reaction mix for 1 hr at 37°C. Slides incubated with the Label Solution that was lacking the terminal transferase enzyme were used as a negative control. Slides were washed for 10 min in PBS three times and mounted with a coverslip.

For the proliferation assay, explants cultured in the presence of C‐CPE or GST for 72 hr were fixed in 10% formalin for 1 hr at room temperature and processed for cryosectioning. Ki67 antibody (ab15580; Abcam) was used at a 1:500 dilution. Alexa Fluor 555 goat anti‐rabbit (A32732; Invitrogen) was used as the secondary antibody at a concentration of 1:500.

### 2.4 Whole mount in situ hybridization

In situ hybridization was performed on E12‐13 kidneys as described ( Nieto, Patel, & Wilkinson, 1996). All kidneys were fixed overnight in 4% PFA in PBS at 4°C. cDNA clones for *Cldn3*, *Cldn4, Cldn6, Cldn7, Cldn8,* and *Cldn14* were generated by RT‐PCR amplification of the coding sequences using E15 mouse kidney total RNA as a template as indicated ( Khairallah et al., 2014). Amplified DNA fragments were sequenced to confirm their identity and then subcloned into the PCRII‐TOPO vector (Invitrogen, Camarillo, CA). To generate antisense riboprobes for in situ hybridization analysis, the mouse claudin cDNA sequences were linearized using Sma1 (*Cldn4*, *Cldn6*, *Cldn8*, *Cldn14*), Xho1 (*Cldn3*), or BamH1 (*Cldn7*) restriction enzymes. In vitro gene transcription was performed using 1 µL T3 (*Cldn3, Cldn4, Cldn6, Cldn8, Cldn14*) or SP6 (*Cldn7*) RNA polymerase. Anti‐DIG antibody conjugated to alkaline phosphatase was used to detect duplexes of DIG‐labeled antisense riboprobe hybridized to *claudin* mRNA sequences. Treated samples were developed using nitro blue tetrazolium chloride, 5‐bromo‐4‐chloro‐3‐indolyl phosphatase substrate in NTMT.

### 2.5 Immunofluorescent detection of claudin proteins

Mouse kidney explants were fixed using 6:3:1 (ethanol: water: 37% PFA). They were then incubated at 4°C overnight with a 1:200 dilution of rabbit anti‐Cldn3 (Invitrogen, 34–700), 1:200 dilution of rabbit anti‐Cldn4 (Invitrogen, 364,800), 1:25 dilution of rabbit anti‐Cldn6 (Abcam, 364,800), 1:100 dilution of rabbit anti‐Cldn7 (Invitrogen, 349,100), or a 1:50 dilution of rabbit anti‐Cldn8 (Invitrogen, 40‐0700Z). R488 and Alexa Fluor 555 goat anti‐rabbit (A11034 and A21127; Invitrogen) were used as secondary antibodies at a concentration of 1/500 for 1 hr at room temperature. A Zeiss LSM780 confocal microscope was used to generate images.

### 2.6 Quantitative and statistical analysis

TUNEL and proliferation assays quantification: Confocal images were analyzed by ImageJ using the following formula. The cellular area representing apoptotic or proliferating cells was detected by red fluorochromes, Alexa Fluor 555 or TMR, respectively, while the total area of the kidney explant was determined by outlining the DAPI positive cells. The ureteric bud area (Total UB area) was delimited using GFP signal that was expressed by the *Hoxb7/GFP* ^+/−^ embryo (Janke, Ward, & Vogel, 2019).

(1) $$\text{Normalized ratio of fluorescence}\;\left(\%\right)=\frac{\text{Area of red fluorescent signals within kidney explant}}{\text{Total area of kidney explant ot Total UB area}}$$

#### 2.6.1 Ureteric bud tip counting

Ureteric bud tips were manually counted as single, bifid and trifid ureteric tips. Lumen tip volume was quantified from confocal images by examining six stacks/kidney. The presence of any black space in the lumen was defined as an enlarged lumen. The percentage of enlarged lumens was derived by the number of enlarged UB tips/total number of UB tips X 100.

#### 2.6.2 Statistical analysis

One‐way ANOVA and post hoc Tukey's multiple comparisons test were used to assess changes in ureteric bud branching. Chi‐square analysis was used to assess the proportion of single, bifid, and trifid ureteric bud tips. The Student's *t* test was used to determine if there were statistically significant differences between two groups for the TUNEL and proliferation data. Data are presented as mean ± standard deviation for all the experiments.

## 3 RESULTS

### 3.1 Removal of C‐CPE‐sensitive claudins decreased ureteric bud branching morphogenesis

Given the large number of claudin family members expressed in the developing mouse kidney ( Khairallah et al., 2014), we utilized a protein reagent that binds to multiple claudins and can simultaneously remove them from tight junctions. The full‐length enterotoxin of *Clostridium perfringens*, (CPE) preferentially binds to Claudin‐3, −4, −6, −7, −8, and −14 and exerts a cytotoxic effect to create pores within epithelial cells that result in epithelial cell death. In contrast, a modified form of the full‐length toxin, containing only the C‐terminal domain of the protein, C‐CPE, is able to remove the same claudins from tight junctions, but without any cytotoxic effects (Fujita et al., 2000; Gao & McClane, 2012; Katahira, Inoue, et al., 1997; Katahira, Sugiyama, et al., 1997; Kimura et al., 2010; Lohrberg et al., 2009; Sonoda et al., 1999; Veshnyakova et al., 2010; Winkler et al., 2009).

**FIGURE 1:**
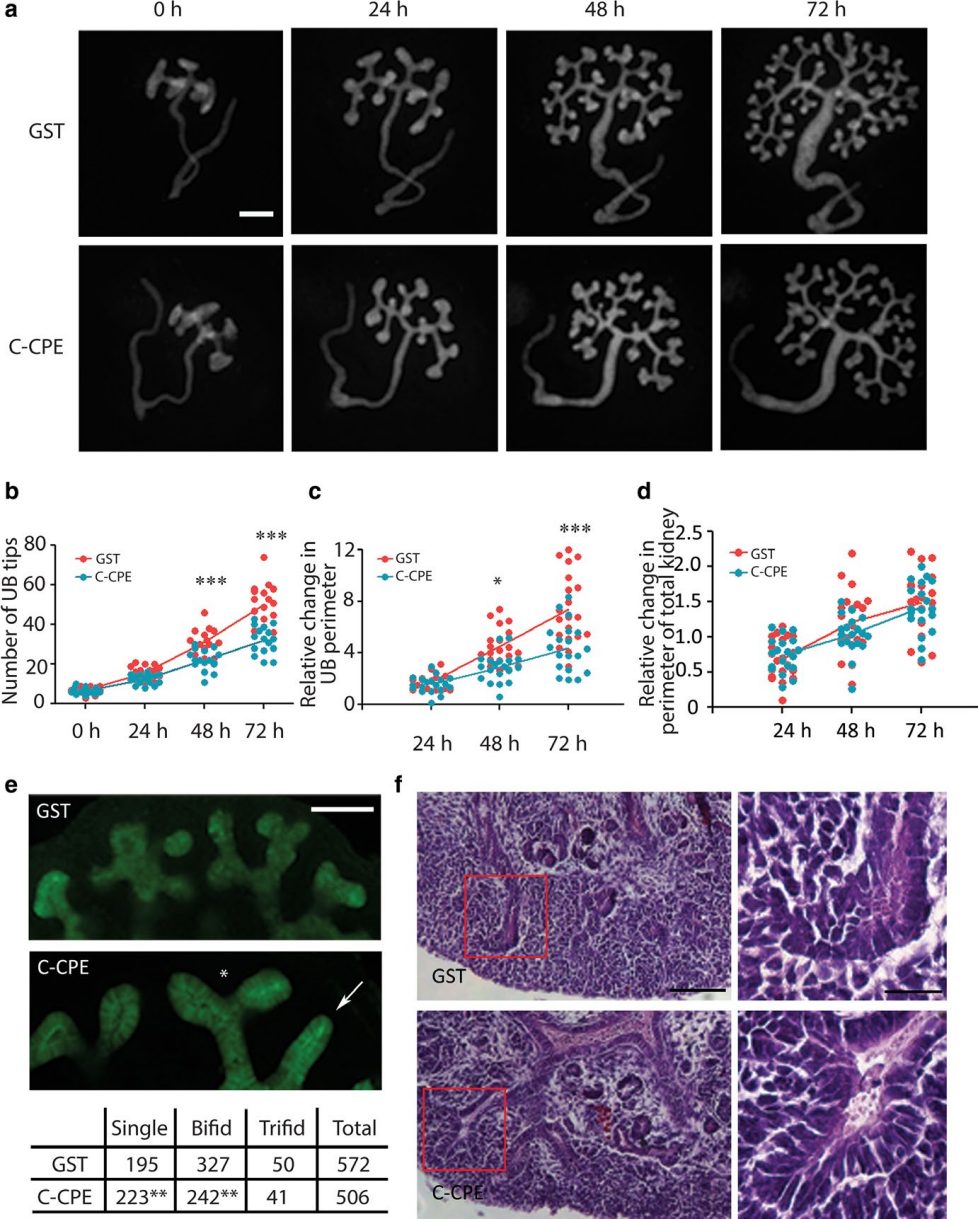
C‐CPE Inhibits Branching Morphogenesis in Mouse Embryonic Kidney Explants. (a) For each embryo at E12, one *Hoxb7/GFP* ^+/−^ kidney explant was grown in GST, and the other was grown in C‐CPE for 72 hr. Scale bar = 200 μm (b–d) Three graphs are shown from left to right that quantify the total number of ureteric bud tips at 24, 48 and 72 hr (red = GST‐treated and blue = C‐CPE‐treated explants) (b) the change in the number of ureteric bud tips, *n* = 17 kidneys in each group; (c) the relative change in ureteric bud perimeter (P) (P_final(f)_ − P_initial(i)_/P_i_) at each time interval, *n* = 19 kidneys in each group (d), the relative change in the total kidney explant perimeter (P_f_ − P_i_/P_i_) at each time interval *n* = 19 for each group. At 48 and 72 hr, there was a significant decrease in the number of ureteric bud tips observed in C‐CPE‐treated compared to GST‐treated explants. Similarly, at 48 and 72 hr, there was a decrease in the relative change in ureteric bud perimeter in C‐CPE‐treated compared to GST‐treated explants. In contrast, the relative change in perimeter of the total kidney explant was similar in GST and C‐CPE‐treated explant suggesting C‐CPE was specifically affecting the ureteric bud compartment. (e) The total number of single, bifid, and trifid ureteric bud tips was enumerated in GST‐ and C‐CPE‐treated kidneys as shown (*n* = 20/group) after 72 hr of culture. There were significantly more single ureteric bud tips and fewer bifid ureteric bud tips in C‐CPE‐treated kidney explants compared to GST, suggesting a decrease in complexity of ureteric bud branching morphogenesis. Scale bar = 100 μm. The asterisk labels an example of a bifid tip, while the arrow shows an example of a single ureteric bud tip in the C‐CPE kidney explant. (f) Representative sections from kidney explants grown for 72 hr that were stained with hematoxylin and eosin are shown. The C‐CPE‐treated explants exhibited ureteric bud tips with enlarged lumens compared to the GST‐treated explants. Red boxes outline the ureteric bud tips shown in the inset images on the right. The inset images show an enlarged ureteric bud tip in the C‐CPE‐treated explant compared to GST. Scale bar = 100 μm. Inset scale bar = 25 μm Statistical analysis one‐way ANOVA and Post hoc Tukey's multiple comparison test for 1b‐d. Chi‐square analysis for 1e. * *p* < .05,** *p* < .01, ***p < .001

We treated mouse embryonic day (E) 12 kidney explants expressing the *Hoxb7/GFP* ^+/−^ transgene, which allowed us to easily monitor ureteric bud growth and branching by the green fluorescent protein expressed in this lineage (Srinivas et al., 1999). To minimize the effects of inter‐embryo variability, one kidney from each embryo was cultured with a GST‐purified form of the C‐CPE, and the other kidney was cultured with GST protein alone for 72 hr. We observed that the formation of ureteric bud (UB) tips was inhibited in the presence of C‐CPE compared to explants grown in the presence of GST alone (Figure 1a). The inhibitory effect on ureteric bud branching was first observed at 24 hr of culture and continued to be present up to 72 hr after culture (*p* < .05 at 24, 48 and 72 hr, Figure 1b). The decrease in ureteric bud tip counts correlated with an overall decrease in the perimeter of the ureteric bud tree in C‐CPE‐treated when compared to GST‐treated explants (Figure 1c). The effect of treatment with C‐CPE appeared to be limited to the ureteric bud lineage since the perimeter of whole kidney explants measured over time in culture was similar in GST and C‐CPE‐treated explants (Figure 1d).

A comparison of the complexity of ureteric bud tips revealed significantly more single UB tips (C‐CPE: 223/506, 44.1% versus GST: 195/572, 34.1% χ^2^ = 11.26, *p* = .007) and significantly less bifid UB tips in C‐CPE‐treated explants (C‐CPE: 242/506, 47.8% versus GST: 327/572, 57.2%, χ^2^ = 9.4, *p* = .002) compared to GST‐treated explants. Similar numbers of trifid UB tips were observed in both groups (C‐CPE: 41/506, 8.1% versus 50/572, GST: 8.7%, χ^2^ = 0.14, *p* = .71) (Figure 1e). A total of 20 kidneys in each group were assessed for the analysis.

To determine if there was any histological evidence of necrosis or toxicity, cultured explants were paraffin‐embedded, sectioned, and then stained with hematoxylin and eosin. These studies revealed no histological evidence of necrosis or toxicity with both the ureteric bud and the mesenchymal lineages intact. The sections suggested there was an increase in lumen volume in ureteric bud tips in C‐CPE‐treated when compared to GST‐treated explants (Figure 1f). Taken together, these data show that C‐CPE specifically inhibited the formation of ureteric bud tips and this correlated with an overall decrease in the perimeter of the ureteric bud lineage.

To determine if the decrease in ureteric bud tip number was due to either an increase in apoptosis or a decrease in proliferation within the UB lineage, TUNEL and Ki‐67 assays were performed on cryosections taken from the cultured explants. These studies revealed that there was no significant difference in the amount of proliferation (mean of total red fluorescent signal expressed as pixels/total UB area expressed as pixels *100 ± standard deviation, GST: 3.07% ± 2.75 versus C‐CPE: 3.35% ± 2.33, *p* = .67) or apoptosis (mean of total red fluorescent signal/total UB area *100 ± standard deviation, GST: 0.84% ±0.85 versus C‐CPE: 0.87% ±0.92, *p* =.87) in the UB lineage in explants grown in the presence of GST or C‐CPE at 72 hr (*n* = 3 kidneys/group and 10 sections/kidney were quantified).

### 3.2 C‐CPE removed claudins from the ureteric bud and increased the lumen of ureteric bud tips

**FIGURE 2:**
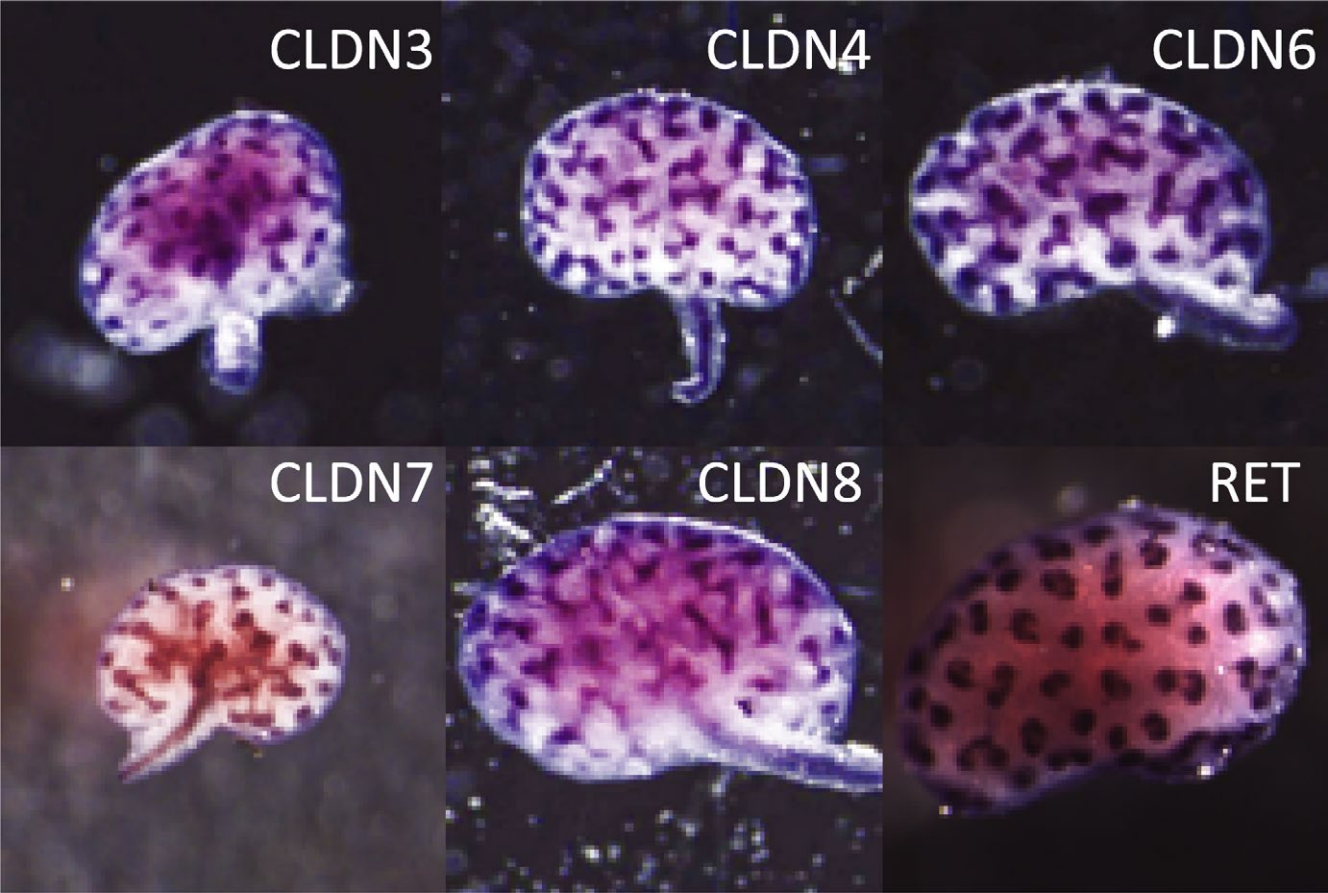
C‐CPE‐Sensitive Claudins Are Detected in Mouse Embryonic Kidneys by Whole Mount In Situ Hybridization. Whole mount in situ hybridization was performed on mouse kidney explants between E12‐13 and revealed the presence of *Cldn3, −4, −6, −7, and −8* transcripts within the ureteric bud tips and trunks. *Cldn14* was not detected by whole mount in situ hybridization and is not shown. Whole mount in situ hybridization to *c‐Ret* is shown as a positive control and its pattern reveals that *c‐Ret* is primarily in the ureteric bud tips with much less signal seen in the trunks in contrast to the C‐CPE‐sensitive claudins. Representative images are shown from a total of three experiments with *n* = 3 kidneys exposed to each ribroprobe

To understand which of the C‐CPE‐sensitive claudins would be targeted by C‐CPE, we characterized their expression patterns during mouse kidney development. By whole mount in situ hybridization, *Cldn3*, −*4, −6, −7,* and *−8* mRNA transcripts were all detected in the branching ureteric bud lineage in both ureteric trunks and ureteric bud tips at embryonic day 12–13 (Figure 2). Claudin‐14 was not detected by whole mount in situ hybridization (data not shown).

**FIGURE 3:**
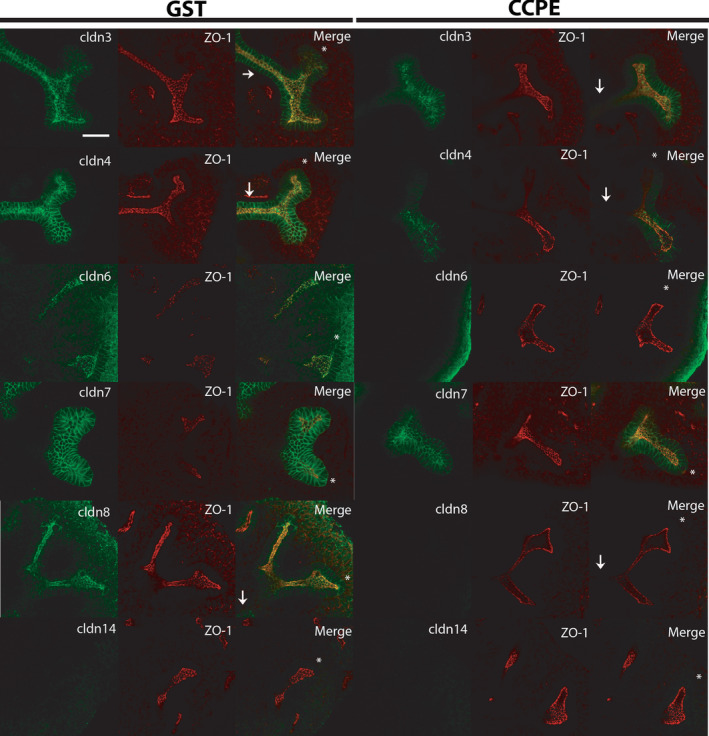
C‐CPE Removes C‐CPE‐Sensitive Claudins from Tight Junctions within the Ureteric Bud Lineage and Increases the Lumens of Ureteric Bud Tips. E12 kidney explants were grown in the presence of GST or C‐CPE and then whole mount immunofluorescence was performed to detect the presence of the C‐CPE‐sensitive claudins and the tight junction marker ZO‐1 after 72 hr of culture. Confocal images reveal that explants cultured in the presence of GST exhibit strong expression of CLDN3,‐4,‐6, and −8 within the apical membrane of the ureteric bud trunks and tips and colocalization with ZO‐1. No signal for CLDN14 protein was detected by immunofluorescence. CLDN7 was predominantly expressed in the basolateral membrane and showed poor colocalization with ZO‐1. In the presence of C‐CPE, there was a marked reduction in signal detected for CLDN3 and −4 in the apical membrane and almost no signal detected for CLDN6 and −8. In contrast, there was relatively more apical expression of CLDN7 following treatment with C‐CPE, but poor colocalization with ZO‐1. Representative images are shown from a total of three experiments with *n* = 3 GST and *n* = 3 C‐CPE‐treated explants in each experiment. The white asterisk marks ureteric bud tips while the white arrow marks the ureteric trunk in the merge images. Scale bar = 50 μm

To determine if C‐CPE‐sensitive claudins were removed from tight junctions in the presence of C‐CPE as predicted, immunofluorescent studies were performed on cultured explants. Control mouse explants grown in the presence of GST alone, showed strong expression of CLDN‐3, −4, −6, and −8 proteins in the apical membrane of the ureteric bud trunks and tips (Figure 3) that colocalized with zona occludens (ZO)‐1, another tight junction protein. In contrast, CLDN‐7 showed high expression in the basolateral membrane and weak signal in the apical membrane with poor colocalization with ZO‐1. CLDN‐14 was not expressed in the ureteric bud lineage by immunofluorescence. In the presence of C‐CPE, there was much less expression of CLDN‐3 and −4 and an even greater loss of CLDN‐6 and CLDN‐8 in the apical membrane of the ureteric bud lineage. Interestingly, in the presence of C‐CPE, CLDN‐7 appeared to show relatively more apical compared to basolateral expression, but again there was poor colocalization with ZO‐1, suggesting CLDN‐7 was not within the tight junction. The immunofluorescent images also suggested that the ureteric bud lumens of C‐CPE‐treated explants were enlarged compared to GST‐treated explants. This was quantified from confocal images and there were significantly more ureteric bud tips with enlarged lumens in C‐CPE‐treated compared to GST‐treated explants (C‐CPE: 35/40, 87.5% versus GST: 10/97, 10.3%, χ^2^ = 66.77, *p * = .0001, *n* = 5 kidneys/group). The enlarged lumens were predominantly seen in ureteric bud tips, but some ureteric trunks also showed this phenotype.

## 4 DISCUSSION

In this study, we demonstrate that claudins are expressed in the ureteric bud lineage during the period of most rapid branching morphogenesis in vivo (Short et al., 2014). Five claudins that are known to bind to the truncated form of the *Clostridium perfringens* enterotoxin (C‐CPE), Claudin‐3, −4, −6, −7, and −8 were expressed in ureteric bud tips and trunks. Embryonic day 12 kidneys that were cultured for 72 hr with C‐CPE exhibited impaired branching morphogenesis with a decrease in the formation of ureteric bud tips. The ureteric bud tips that formed in the presence of C‐CPE had larger lumens and were more likely to exist as single rather than bifid tips, suggesting an overall decrease in the complexity of branching. The decrease in ureteric bud tips from treatment with C‐CPE correlated with a decrease in expression and co‐localization with ZO‐1 for all of the C‐CPE‐sensitive claudins with the exception of CLDN‐7, so that they were absent or weakly expressed in the apical membrane. Taken together, claudins are required for ureteric bud branching morphogenesis and regulate the formation of ureteric bud tip lumens.

We removed multiple claudins using the C‐CPE reagent because removal of single C‐CPE‐sensitive claudins has not generated severe renal developmental phenotypes, suggesting there may be functional redundancy amongst members and/or different requirements depending on the model organism. For example, removal of Claudin‐3, −4, −6, or −8 has not led to any defect in mouse kidney development based on the fact that the offspring survive and do not succumb to renal failure postnatally (Anderson et al., 2008; Castro Dias et al., 2019; Fujita, Hamazaki, Noda, Oshima, & Minato, 2012; Gong et al., 2015). In the absence of comprehensive counts of nephron number, however, we cannot rule out the possibility that the mice may have mild to moderate defects in nephron number from a deficiency in renal branching morphogenesis. Indeed, we performed nephron number counts on Claudin‐7 knockout mice, which die at 2 weeks of age, but we did not observe any difference between mutants and wild‐type littermates (Khairallah et al., 2014). In *Xenopus,* however, knockdown of Claudin‐6 did result in decreased apical‐basal polarity and cell adhesion within the pronephric tubule (Sun, Wang, Li, & Mao, 2015).

**FIGURE 4:**
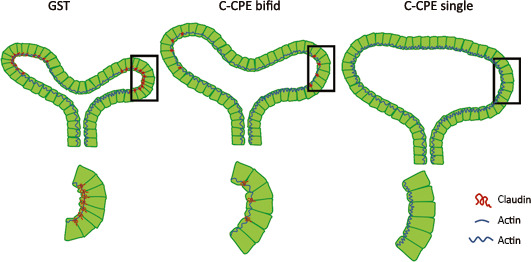
Proposed Mechanism by which Claudins Regulate Branching Morphogenesis. Claudins are normally localized to tight junctions within the apical membrane where they may create a tensile force through their interaction with the actin cytoskeleton that maintains the shape of the ureteric bud lineage and its individual cells. In the presence of C‐CPE, specific C‐CPE‐sensitive claudins are removed from the apical membrane. We speculate that this results in a decrease in tensile force along the apical membrane that results in larger lumens within ureteric bud tips and less efficient branching such that there is a propensity to see more single and bifid ureteric bud tips in the presence of C‐CPE. The decision for greater arborization, for example, bifid versus trifid ureteric bud tips may be due to local effects on tensile force along the apical membrane that are regulated by the relative expression of claudins within ureteric bud cells

A number of studies have demonstrated that claudins are expressed within the ureteric bud and its derivatives during kidney development at a time when there is no urine filtrate formed by glomerular filtration (Wang et al., 2011). This begs the question, what is their function at this timepoint when there is no significant paracellular transport? In our studies, the decrease in branching morphogenesis following claudin removal was not due to a change in cell proliferation or in apoptosis within the ureteric bud lineage. We did observe larger lumens in the ureteric bud tips of C‐CPE‐treated kidneys and less complexity of branching morphogenesis compared to GST controls. We speculate that claudins within the ureteric bud lineage maintain tensile forces at the apical membrane through their interactions with the actin cytoskeleton and that these tensile forces are maximally taut where new ureteric buds will arise (Figure 4). Analogous to the purse‐string hypothesis, high tensile forces at the apical membrane would alter the shape of individual cells to a wedge as opposed to a columnar shape, and this would confer the emergence of a bud from the ureteric bud epithelium. After binding to C‐CPE, C‐CPE‐sensitive claudins (CLDN3, −4, −6, and −8) are removed from tight junctions and this is predicted to decrease tension along the apical membrane. This would cause a decrease in the number of wedge‐shaped cells at the ureteric bud tips that would favor larger ureteric bud tip lumens and less bud formation. Tight junctions are highly dynamic structures based on in vitro cell culture models (Shen, Weber, & Turner, 2008). In fibroblasts, claudins associate intermittently with ZO‐1 and actin and this promotes the formation of tight junction strands between cells. However, claudins can also anneal or break from strands independently of ZO‐1 and actin. Indeed, it is hypothesized that the ability of tight junction strands to form independently of ZO‐1 and actin permits the epithelium to separate its permeability properties from its interactions with the cytoskeleton via tight junction complexes. In summary, we believe that claudins may function at this stage of branching morphogenesis to regulate cell shape through their effects on the tensile forces along the apical membrane.

While we know much about the signaling factors and transcription factors that drive branching morphogenesis, we still have an incomplete understanding of the cellular events that drive this process. Cell proliferation and mitosis‐associated dispersal occurs within the ureteric bud lineage during branching, but these do not seem to be the major processes that drive the formation of new ureteric bud tips. Our studies in which we have removed specific claudin members from the tight junctions clearly affects the number and complexity of ureteric bud tips that form, but branching still occurs, albeit less efficiently. To address the role of claudins during renal branching morphogenesis, in vivo models will need to be established in which multiple C‐CPE‐sensitive claudins are removed. The development of such a model will permit complete removal of claudins as opposed to partial removal as observed in our explant experiments.

## References

[phy214492-bib-0001] Anderson, W. J. , Zhou, Q. , Alcalde, V. , Kaneko, O. F. , Blank, L. J. , Sherwood, R. I. , … Melton, D. A. (2008). Genetic targeting of the endoderm with claudin‐6CreER. Developmental Dynamics, 237, 504–512. 10.1002/dvdy.21437 18213590 PMC2665265

[phy214492-bib-0002] Baumholtz, A. I. , Simard, A. , Nikolopoulou, E. , Oosenbrug, M. , Collins, M. M. , Piontek, A. , … Ryan, A. K. (2017). Claudins are essential for cell shape changes and convergent extension movements during neural tube closure. Developmental Biology, 428, 25–38. 10.1016/j.ydbio.2017.05.013 28545845 PMC5523803

[phy214492-bib-0003] Castro Dias, M. , Coisne, C. , Lazarevic, I. , Baden, P. , Hata, M. , Iwamoto, N. , … Engelhardt, B. (2019). Claudin‐3‐deficient C57BL/6J mice display intact brain barriers. Scientific Reports, 9, 203 10.1038/s41598-018-36731-3 30659216 PMC6338742

[phy214492-bib-0004] Costantini, F. (2012). Genetic controls and cellular behaviors in branching morphogenesis of the renal collecting system. Wiley Interdisciplinary Reviews: Developmental Biology, 1, 693–713. 10.1002/wdev.52 22942910 PMC3430146

[phy214492-bib-0005] Fujita, H. , Hamazaki, Y. , Noda, Y. , Oshima, M. , & Minato, N. (2012). Claudin‐4 deficiency results in urothelial hyperplasia and lethal hydronephrosis. PLoS One, 7, e52272 10.1371/journal.pone.0052272 23284964 PMC3528782

[phy214492-bib-0006] Fujita, K. , Katahira, J. , Horiguchi, Y. , Sonoda, N. , Furuse, M. , & Tsukita, S. (2000). Clostridium perfringens enterotoxin binds to the second extracellular loop of claudin‐3, a tight junction integral membrane protein. FEBS Letters, 476, 258–261. 10913624 10.1016/s0014-5793(00)01744-0

[phy214492-bib-0007] Gao, Z. , & McClane, B. A. (2012). Use of clostridium perfringens enterotoxin and the enterotoxin receptor‐binding domain (C‐CPE) for cancer treatment: Opportunities and challenges. Journal of Toxicology, 2012, 981626. 21941545 10.1155/2012/981626 PMC3173885

[phy214492-bib-0008] Gong, Y. , Wang, J. , Yang, J. , Gonzales, E. , Perez, R. , & Hou, J. (2015). KLHL3 regulates paracellular chloride transport in the kidney by ubiquitination of claudin‐8. Proceedings of the National Academy of Sciences, USA, 112, 4340–4345. 10.1073/pnas.1421441112 PMC4394310 25831548

[phy214492-bib-0009] Gupta, I. R. , Lapointe, M. , & Yu, O. H. (2003). Morphogenesis during mouse embryonic kidney explant culture. Kidney International, 63, 365–376. 10.1046/j.1523-1755.2003.00715.x 12472805

[phy214492-bib-0010] Haddad, N. , El Andalousi, J. , Khairallah, H. , Yu, M. , Ryan, A. K. , & Gupta, I. R. (2011). The tight junction protein claudin‐3 shows conserved expression in the nephric duct and ureteric bud and promotes tubulogenesis in vitro. American Journal of Physiology. Renal Physiology, 301, F1057–1065. 10.1152/ajprenal.00497.2010 21775479

[phy214492-bib-0011] Janke, L. J. , Ward, J. M. , & Vogel, P. (2019). Classification, scoring, and quantification of cell death in tissue sections. Veterinary Pathology, 56, 33–38. 10.1177/0300985818800026 30278838

[phy214492-bib-0012] Katahira, J. , Inoue, N. , Horiguchi, Y. , Matsuda, M. , & Sugimoto, N. (1997). Molecular cloning and functional characterization of the receptor for *Clostridium perfringens* enterotoxin. Journal of Cell Biology, 136, 1239–1247. 10.1083/jcb.136.6.1239 9087440 PMC2132509

[phy214492-bib-0013] Katahira, J. , Sugiyama, H. , Inoue, N. , Horiguchi, Y. , Matsuda, M. , & Sugimoto, N. (1997). Clostridium perfringens enterotoxin utilizes two structurally related membrane proteins as functional receptors in vivo. Journal of Biological Chemistry, 272, 26652–26658. 9334247 10.1074/jbc.272.42.26652

[phy214492-bib-0014] Khairallah, H. , El Andalousi, J. , Simard, A. , Haddad, N. , Chen, Y.‐H. , Hou, J. , … Gupta, I. R. (2014). Claudin‐7, ‐16, and ‐19 during mouse kidney development. Tissue Barriers, 2, e964547 10.4161/21688362.2014.964547 25610756 PMC4292044

[phy214492-bib-0015] Kimura, J. , Abe, H. , Kamitani, S. , Toshima, H. , Fukui, A. , Miyake, M. , … Horiguchi, Y. (2010). Clostridium perfringens enterotoxin interacts with claudins via electrostatic attraction. Journal of Biological Chemistry, 285, 401–408. 19903817 10.1074/jbc.M109.051417 PMC2804187

[phy214492-bib-0016] Lohrberg, D. , Krause, E. , Schümann, M. , Piontek, J. , Winkler, L. , Blasig, I. E. , & Haseloff, R. F. (2009). A strategy for enrichment of claudins based on their affinity to *Clostridium perfringens* enterotoxin. BMC Molecular Biology, 10, 61 10.1186/1471-2199-10-61 19545418 PMC2713237

[phy214492-bib-0017] Menshykau, D. , Michos, O. , Lang, C. , Conrad, L. , McMahon, A. P. , & Iber, D. (2019). Image‐based modeling of kidney branching morphogenesis reveals GDNF‐RET based Turing‐type mechanism and pattern‐modulating WNT11 feedback. Nature Communications, 10, 239 10.1038/s41467-018-08212-8 PMC6484223 30651543

[phy214492-bib-0018] Michael, L. , & Davies, J. A. (2004). Pattern and regulation of cell proliferation during murine ureteric bud development. Journal of Anatomy, 204, 241–255. 10.1111/j.0021-8782.2004.00285.x 15061751 PMC1571296

[phy214492-bib-0019] Moriwaki, K. , Tsukita, S. , & Furuse, M. (2007). Tight junctions containing claudin 4 and 6 are essential for blastocyst formation in preimplantation mouse embryos. Developmental Biology, 312, 509–522. 10.1016/j.ydbio.2007.09.049 17980358

[phy214492-bib-0020] Nieto, M. A. , Patel, K. , & Wilkinson, D. G. (1996). In situ hybridization analysis of chick embryos in whole mount and tissue sections. Methods in Cell Biology, 51, 219–235. 8722478 10.1016/s0091-679x(08)60630-5

[phy214492-bib-0021] Packard, A. , Georgas, K. , Michos, O. , Riccio, P. , Cebrian, C. , Combes, A. N. , … Costantini, F. (2013). Luminal mitosis drives epithelial cell dispersal within the branching ureteric bud. Developmental Cell, 27, 319–330. 10.1016/j.devcel.2013.09.001 24183650 PMC3926506

[phy214492-bib-0022] Schmidt‐Ott, K. M. , Yang, J. , Chen, X. , Wang, H. , Paragas, N. , Mori, K. , … Barasch, J. (2005). Novel regulators of kidney development from the tips of the ureteric bud. Journal of the American Society of Nephrology, 16, 1993–2002. 10.1681/ASN.2004121127 15917337

[phy214492-bib-0023] Shen, L. , Weber, C. R. , & Turner, J. R. (2008). The tight junction protein complex undergoes rapid and continuous molecular remodeling at steady state. Journal of Cell Biology, 181, 683–695. 10.1083/jcb.200711165 18474622 PMC2386107

[phy214492-bib-0024] Short, K. M. , Combes, A. N. , Lefevre, J. , Ju, A. L. , Georgas, K. M. , Lamberton, T. , … Little, M. H. (2014). Global quantification of tissue dynamics in the developing mouse kidney. Developmental Cell, 29, 188–202. 10.1016/j.devcel.2014.02.017 24780737

[phy214492-bib-0025] Siddiqui, A. S. , Khattra, J. , Delaney, A. D. , Zhao, Y. , Astell, C. , Asano, J. , … Marra, M. A. (2005). A mouse atlas of gene expression: Large‐scale digital gene‐expression profiles from precisely defined developing C57BL/6J mouse tissues and cells. Proceedings of the National Academy of Sciences, USA, 102, 18485–18490. 10.1073/pnas.0509455102 PMC1311911 16352711

[phy214492-bib-0026] Sonoda, N. , Furuse, M. , Sasaki, H. , Yonemura, S. , Katahira, J. , Horiguchi, Y. , & Tsukita, S. (1999). Clostridium perfringens enterotoxin fragment removes specific claudins from tight junction strands: Evidence for direct involvement of claudins in tight junction barrier. Journal of Cell Biology, 147, 195–204. 10508866 10.1083/jcb.147.1.195 PMC2164970

[phy214492-bib-0027] Srinivas, S. , Goldberg, M. R. , Watanabe, T. , D'Agati, V. , & al‐ Awqati Costantini F. (1999). Expression of green fluorescent protein in the ureteric bud of transgenic mice: A new tool for the analysis of ureteric bud morphogenesis. Developmental Genetics, 24, 241–251. 10322632 10.1002/(SICI)1520-6408(1999)24:3/4<241::AID-DVG7>3.0.CO;2-R

[phy214492-bib-0028] Stuart, R. O. , Bush, K. T. , & Nigam, S. K. (2003). Changes in gene expression patterns in the ureteric bud and metanephric mesenchyme in models of kidney development. Kidney International, 64, 1997–2008. 10.1046/j.1523-1755.2003.00383.x 14633122

[phy214492-bib-0029] Sun, J. , Wang, X. , Li, C. , & Mao, B. (2015). Xenopus Claudin‐6 is required for embryonic pronephros morphogenesis and terminal differentiation. Biochemical and Biophysical Research Communications, 462, 178–183. 10.1016/j.bbrc.2015.04.065 25979361

[phy214492-bib-0030] Veshnyakova, A. , Protze, J. , Rossa, J. , Blasig, I. E. , Krause, G. , & Piontek, J. (2010). On the interaction of *Clostridium perfringens* enterotoxin with claudins. Toxins, 2, 1336–1356. 10.3390/toxins2061336 22069641 PMC3153257

[phy214492-bib-0031] Wang, H. , Li, Q. , Liu, J. , Mendelsohn, C. , Salant, D. J. , & Lu, W. (2011). Noninvasive assessment of antenatal hydronephrosis in mice reveals a critical role for Robo2 in maintaining anti‐reflux mechanism. PLoS One, 6, e24763 10.1371/journal.pone.0024763 21949750 PMC3176762

[phy214492-bib-0032] Winkler, L. , Gehring, C. , Wenzel, A. , Müller, S. L. , Piehl, C. , Krause, G. , … Piontek, J. (2009). Molecular determinants of the interaction between *Clostridium perfringens* enterotoxin fragments and claudin‐3. Journal of Biological Chemistry, 284, 18863–18872. 19429681 10.1074/jbc.M109.008623 PMC2707212

